# Plasma miRNA-146b-3p, -222-3p, -221-5p, -21a-3p Expression Levels and *TSHR* Methylation: Diagnostic Potential and Association with Clinical and Pathological Features in Papillary Thyroid Cancer

**DOI:** 10.3390/ijms25158412

**Published:** 2024-08-01

**Authors:** Mintaute Kazlauskiene, Raimonda Klimaite, Aiste Kondrotiene, Albertas Dauksa, Dalia Dauksiene, Rasa Verkauskiene, Birute Zilaitiene

**Affiliations:** 1 Institute of Endocrinology, Medical Academy, Lithuanian University of Health Sciences, 50161 Kaunas, Lithuania; mintaute.kazlauskiene@lsmuni.lt (M.K.); aiste.kondrotiene@lsmuni.lt (A.K.); dalia.dauksiene@lsmuni.lt (D.D.); rasa.verkauskiene@lsmuni.lt (R.V.); birute.zilaitiene@lsmuni.lt (B.Z.); 2 Institute of Digestive Research, Faculty of Medicine, Medical Academy, Lithuanian University of Health Sciences, 50161 Kaunas, Lithuania; albertas.dauksa@lsmuni.lt

**Keywords:** papillary thyroid cancer, miRNA-222-3p, miRNA-146b-3p, miRNA-221-5p, miRNA-21a-3p, *TSHR* methylation, plasma samples

## Abstract

This study aimed to investigate the expression of microRNAs (miRNAs) -146b-3p, -221-5p, -222-3p, and -21a-3p and the methylation pattern of the thyroid-stimulating hormone receptor (*TSHR*) gene in blood plasma samples from papillary thyroid cancer (PTC) patients before and after thyroidectomy compared to healthy controls (HCs). This study included 103 participants, 46 PTC patients and 57 HCs, matched for gender and age. Significantly higher preoperative expression levels of miRNAs and *TSHR* methylation were determined in the PTC patients compared to HCs. Post-surgery, there was a notable decrease in these biomarkers. Elevated *TSHR* methylation was linked to larger tumor sizes and lymphovascular invasion, while increased miRNA-222-3p levels correlated with multifocality. Receiver operating characteristic (ROC) analysis showed AUCs below 0.8 for all candidate biomarkers. However, significant changes in the expression of all analyzed miRNAs and *TSHR* methylation levels indicate their potential to differentiate PTC patients from healthy individuals. These findings suggest that miRNAs and *TSHR* methylation levels may serve as candidate biomarkers for early diagnosis and monitoring of PTC, with the potential to distinguish PTC patients from healthy individuals. Further research is needed to validate these biomarkers for clinical application.

## 1. Introduction

Papillary thyroid cancer (PTC) is the most common type of thyroid cancer, comprising 80–85% of all cases, and its incidence has been rising globally. PTC generally has a good prognosis, with a 10-year survival rate exceeding 90% for most patients [1,2]. However, this can vary based on factors such as tumor size, the extent of disease at diagnosis, the age of the patient, the presence of distant metastases, and histological subtypes [3]. Approximately 10% of cases may have metastatic disease at initial presentation [4,5]. 

Fine-needle aspiration biopsy (FNAB) is the gold standard for evaluating thyroid nodules, yet it has limitations. The sensitivity and specificity of FNAB can vary due to nodule size and location, and the expertise of cytopathologists. Sometimes, results may be inconclusive, necessitating repeat biopsies or additional testing [5]. PTC includes various histological subtypes, each with unique characteristics, requiring a multidisciplinary diagnostic approach. Advances in imaging technology, molecular testing, and risk stratification algorithms enhance the accuracy of PTC diagnosis and reduce unnecessary interventions for low-risk lesions [6]. Postoperatively, serum thyroglobulin (Tg) testing is used to monitor PTC recurrence, though anti-Tg antibodies, present in 10% of healthy individuals and 20–25% of PTC patients, can affect test sensitivity and specificity [5,6]. Biomarkers, imaging studies, and clinical parameters support comprehensive PTC evaluation and management. Integrating biomarker data into risk stratification algorithms aids personalized treatment decisions and improves patient outcomes. 

The thyroid-stimulating hormone receptor (*TSHR*) is found on thyroid cells and encoded on chromosome 14q31.1 by a gene with 12 exons [7]. Epigenetic research shows *TSHR*’s role in thyroid cancer, highlighting its promoter region’s CpG dinucleotides. Changes in DNA methylation can silence the *TSHR* gene in tumors, promoting cancer growth [8]. *TSHR* signaling initiates carcinogenesis and cytokine production in tumor cells, influencing the tumor environment [9,10]. Methylation of gene promoter regions in tumor-specific genes is a potential epigenetic biomarker for cancer detection [11,12]. 

MicroRNAs (miRNAs) are endogenous, non-coding RNA molecules that range from 19 to 25 nucleotides in length and function as post-transcriptional negative regulators of gene expression by binding to the 3′ untranslated region (UTR) of target messenger RNAs (mRNAs) in the cytoplasm [13]. These miRNAs are implicated in fundamental biological processes such as metabolism, cell cycle regulation, tissue differentiation, embryogenesis, and organogenesis. They demonstrate their critical roles in modulating oncogenes and tumor suppressor genes [14]. Extensive research has focused on changes in miRNA expression across various diseases and cancer types [15]. Notably, miRNAs are highly stable in tissues and biofluids (e.g., plasma and serum), and specific miRNAs are released into circulation during disease progression, thus providing a basis for non-invasive biomarker discovery [16]. Recent studies have recognized the hormone-like activity of circulating miRNAs over long distances, although it remains challenging to elucidate their specific roles and implications in particular pathways [17]. In the context of PTC, several miRNAs, including miRNA-146b-3p, miRNA-21a-3p, miRNA-221-5p, and miRNA-222-3p, are known to be dysregulated [18]. These miRNAs have shown potential as biomarkers in both tissue and plasma/serum studies [18,19,20,21,22,23] and are considered potential biomarkers of PTC. 

DNA methylation and miRNAs, though distinct, are interconnected. miRNAs can influence the genes involved in DNA methylation, like DNMTs, affecting the methylation status of genes such as *TSHR* [24,25]. Conversely, changes in methylation can alter miRNA expression [26]. In cancer, the dysregulation of both DNA methylation and miRNAs can coordinate, influencing the complex regulatory networks driving tumorigenesis [24,25]. Hypermethylation of *TSHR* may be associated with specific miRNA dysregulation, affecting the development and progression of thyroid cancer [27,28].

Focus is placed on miRNAs rather than coding genes due to their regulatory potential, which affects multiple genes and broad cellular processes and makes them attractive for research and therapeutic intervention [29,30,31]. miRNAs serve as stable biomarkers in body fluids for cancer diagnosis and prognosis, and for monitoring therapy response [32,33]. Targeting miRNAs with mimics or inhibitors offers a novel approach to cancer treatment [34]. We focused on these miRNAs and *TSHR* methylation due to their significant roles in the pathogenesis of thyroid cancer [35]. These miRNAs are crucial in the regulatory networks influencing cancer cell behavior, with their dysregulation being a hallmark of thyroid cancer [36]. *TSHR* methylation is a vital epigenetic mechanism impacting the development of thyroid cancer [37]. These factors provide a comprehensive understanding of molecular alterations in thyroid cancer and offer potential diagnostic and therapeutic avenues.

Overall, while the potential of miRNAs and *TSHR* methylation as diagnostic and prognostic biomarkers in PTC is evident, further research is required to fully elucidate their specific roles and improve their sensitivity and specificity as clinical tools, especially considering the high costs of ThyroSeq, ThyGeNEXT/ThyraMIR, and ThyroidPrint tests [23]. This study aimed to investigate the differences between the methylation status of *TSHR* and miRNA -222-3p, -221-5p, -21a-3p, and -146b-3p in the blood plasma of individuals diagnosed with papillary thyroid carcinoma (PTC) both pre- and post-surgery. We also sought to establish associations between the methylation levels of *TSHR*; miRNA-222-3p, -221-3p, -21a-3p, and -146b-3p; and various clinicopathological characteristics while exploring their diagnostic significance in PTC.

## 2. Results

### 2.1. Study Population

A total of 103 subjects were included in the prospective part of this study. A total of 46 patients with a histologically confirmed diagnosis of PTC after surgical treatment and 57 healthy controls (HCs) were enrolled. The cases and controls were matched based on their gender and age.

The demographic and clinicopathological characteristics of the study population with PTC and the HCs are shown in Table 1. The majority of patients were diagnosed with pT1a (32.6%) and pT3a (32.6%), unifocal PTC (71.7%), and presented with the classical variant (39.1%). Half of the patients exhibited lymphovascular invasion (54.3%) of PTC. 

### 2.2. Expression of miRNA-146b-3p, -222-3p, -21a-3p, and -221-5p and Methylation of TSHR in Plasma Samples of Patients with PTC and HCs

**Figure 1:**
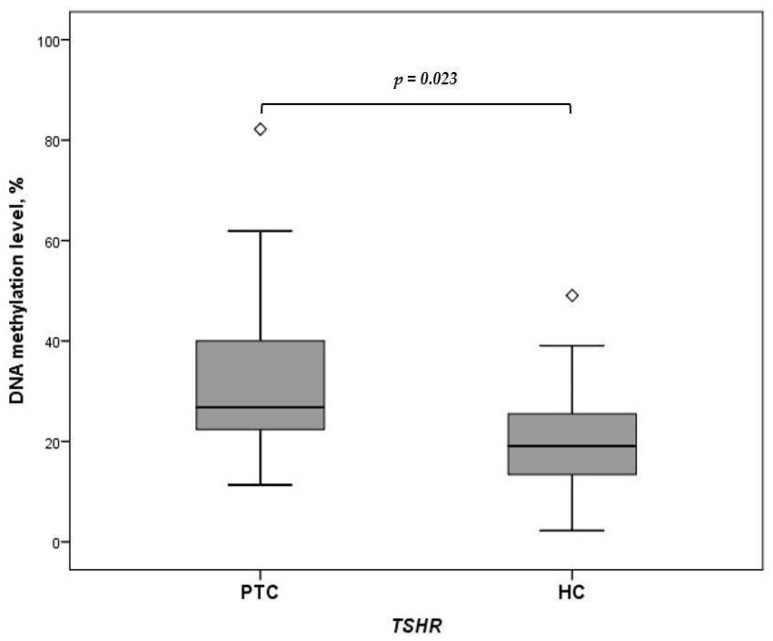
Comparison of *TSHR* methylation levels in plasma samples of PTC patients versus HCs. The Mann–Whitney U test was used to analyze the differences in quantitative traits between the groups.

Expression of miRNA-146b-3p, miRNA-222-3p, miRNA-21a-3p, and miRNA-221-5p was significantly higher in the preoperative PTC group compared to the HC group (*p* < 0.009, *p* < 0.001, *p* < 0.001, *p* = 0.003, respectively) (Table 2). The *TSHR* methylation levels were significantly higher in PTC patients’ plasma than in HCs’ (26.041% vs. 21.382%, *p* = 0.023) (Figure 1).

### 2.3. Plasma miRNA Expression and TSHR Methylation Levels in Patients with PTC before and after Thyroidectomy

The plasma expression levels of miRNA-146b-3p, miRNA-222-3p, miRNA-21a-3p, miRNA-221-5p, and *TSHR* methylation were evaluated post-surgery. There was a significant decrease in the plasma expression of miR-146b-3p, miR-21a-3p, miR-221-5p, and *TSHR* methylation levels post-surgery (*p* = 0.009, <0.001, 0.003, <0.001, and <0.001, respectively) (Table 3). 

**Figure 2:**
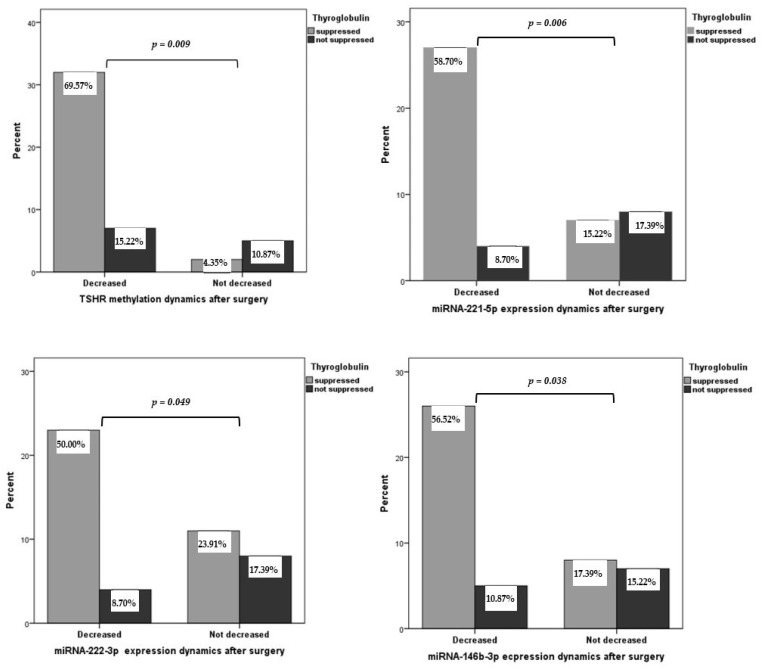
Comparison of *TSHR* methylation levels and miRNA-221-5p, miRNA-222-3p, and miRNA-146b-3p expression dynamics after surgery in PTC patients with suppressed and non-suppressed thyroglobulin. *p* < 0.001. A chi-square independence test was used to determine the differences between the groups.

We evaluated the association of postoperative changes in Tg with *TSHR* methylation and miRNA expression levels. After surgery, the methylation levels of *TSHR* and the expression of miRNA-221-5p, miRNA-222-3p, and miRNA-146b-3p significantly decreased in PTC patients with suppressed Tg concentrations (*p* = 0.009, *p* = 0.006, *p* = 0.049, and *p* = 0.038, respectively) (Figure 2). 

### 2.4. Association of TSHR Methylation Level and miRNA-221-5p, miRNA-21a-3p, miRNA-222-3p, and miRNA-146b-3p Expression with Clinicopathological Features of PTC

We conducted an analysis of plasma *TSHR* methylation and miRNA expression using the demographic and clinicopathological characteristics of the study population. The *TSHR* methylation levels were significantly higher in females with PTC compared to the male group (*p* = 0.038). Patients with larger tumor size (>2 cm) and lymphovascular invasion had significantly higher methylation levels of *TSHR* (*p* = 0.002 and *p* = 0.039, respectively). Our findings indicated that the plasma expression levels of miRNA-222-3p were significantly elevated in multifocal PTC compared to unifocal PTC (*p* = 0.016). Patients with PTC lymphovascular invasion had significantly lower expression levels of miRNA-146b-3p and miRNA-21a-3p (*p* = 0.005 and *p* = 0.007, respectively). The plasma expression levels of miRNA-21a-3p were significantly higher in cases of extrathyroidal extension PTC (*p* = 0.008) (Table 4).

### 2.5. Correlation of miRNA Expression with TSHR Methylation Levels

**Figure 3:**
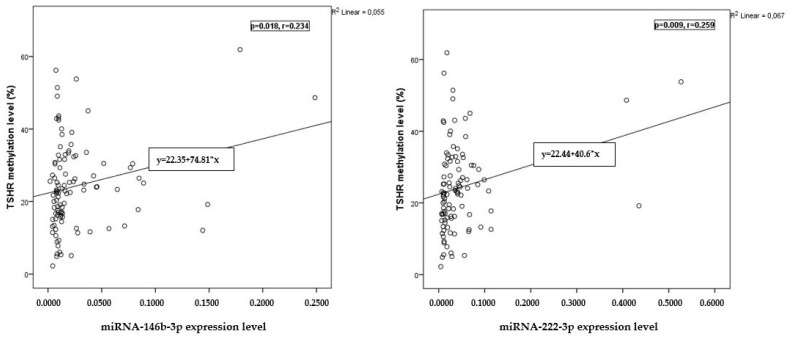
Correlation between *TSHR* methylation and miRNA-146b-3p and miRNA-222-3p expression levels before surgery. The Pearson correlation coefficient was used to measure the strength of a linear association between two variables.

**Figure 4:**
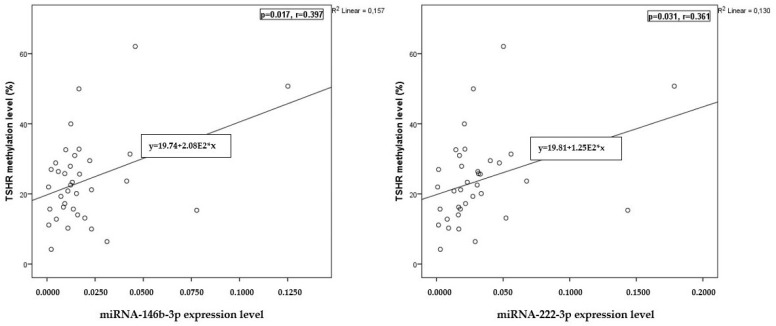
Correlation between *TSHR* methylation and miRNA-146b-3p and miRNA-222-3p expression levels after surgery. The Pearson correlation coefficient was used to measure the strength of a linear association between two variables.

The analysis showed a weak positive correlation between the *TSHR* methylation levels and the expression levels of miRNA-146b-3p and miRNA-222-3p before surgery (*p* = 0.018, r = 0.234 and *p* = 0.009, r = 0.259, respectively) (Figure 3). Moreover, our findings indicated a weak positive correlation between the *TSHR* methylation levels and the expression levels of miRNA-146b-3p and miRNA-222-3p after surgery (*p* = 0.017, r = 0.397 and *p* = 0.031, r = 0.361, respectively) (Figure 4).

### 2.6. The Value of miRNA-146b-3p, -222-3p, -21a-3p, -221-5p, and TSHR Methylation Assays in PTC Plasma Samples for Diagnosis of PTC

**Figure 5:**
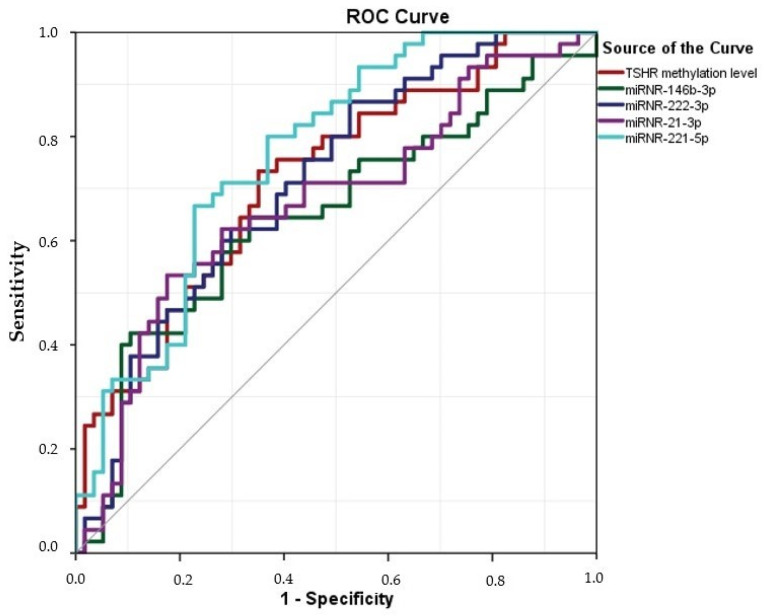
The diagnostic significance of miRNAs and *TSHR* in distinguishing between PTC and HC. ROC curves were used to distinguish the groups. Plasma miRNA-146b-3p, -222-3p, -21a-3p, -221-5p and *TSHR* methylation levels in PTC vs. HC.

ROC analysis was used to assess the diagnostic value of five circulating miRNAs (miRNA-146b-3p, miRNA-222-3p, miRNA-21a-3p, and miRNA-221-5p) and *TSHR* methylation levels for distinguishing between PTC patients and healthy individuals. The results indicated that all investigated miRNAs had statistically significant diagnostic value. Among them, miRNA-221-5p had the highest AUC of 0.765 (95% CI = 0.674–0.855), with a sensitivity of 87.0% and specificity of 50.0% at the cut-off value of 0.05. The *TSHR* methylation levels were statistically significant and satisfactory to differentiate PTC patients from HCs (*p* < 0.0001). *TSHR* had the highest AUC of 0.719 (95% CI = 0.620–0.818), with 86.7% sensitivity and 63.2% specificity (Table 5, Figure 5).

## 3. Discussion

This study analyzed the expression levels of four miRNAs (miRNA-146b-3p, miRNA-21a-3p, miRNA-221-5p, and miRNA-222-3p)—alongside the methylation status of the promoter region of *TSHR*—to evaluate their potential as non-invasive biomarkers for PTC. We conducted a comparative analysis of their expression profiles in plasma samples from PTC patients and HC groups and evaluated their association with the clinicopathological features of PTC. Our findings revealed that all the analyzed biomarkers exhibited significantly elevated levels in PTC patients compared to healthy individuals. Specifically, the expression levels of miRNA-146b-3p, miRNA-222-3p, and miRNA-221-5p and the methylation levels of *TSHR* were observed to decrease post-thyroidectomy in PTC patients with suppressed thyroglobulin (Tg) concentrations. The *TSHR* methylation levels were significantly higher in females with PTC than in males. Patients with larger tumors (>2 cm) and lymphovascular invasion exhibited significantly higher *TSHR* methylation levels. Additionally, our study revealed that plasma levels of miRNA-222-3p were significantly elevated in cases of multifocal PTC compared to unifocal PTC. ROC analysis demonstrated an AUC of <0.8 for all investigated candidate biomarkers; however, the changes in expression for all the analyzed miRNAs and for *TSHR* methylation were statistically significant, thereby indicating their potential in distinguishing PTC patients from healthy individuals.

A worldwide search for reliable PTC biomarkers has resulted in a wide range of genetic classifiers that might be used in diagnosing PTC, such as Afirma GSG, ThyroSec v3, ThyGeNEXT/ThyraMIR, and Thyroid Print. However, they are not widely used in daily practice due to their cost [38]. A simpler, more accessible, cost-effective approach would be extremely useful [39]. Promising epigenetic molecular markers are being widely explored. Previous studies indicate that the levels of miRNA-146b-3p, miRNA-222-3p, miRNA-221-5p, and miRNA-21a-3p are elevated in plasma samples of PTC patients compared to healthy individuals [39,40]. *TSHR* methylation levels are another epigenetic potential biomarker of PTC [12]. To our knowledge, we are the first to investigate miRNA-146b-3p, -222-3p, -21a-3p, and -221-5p and *TSHR* methylation levels in the same population. The significantly higher expression of all four miRNAs and the higher *TSHR* methylation levels in our PTC cohort of patients compared to HCs, strengthened by the high AUC in the ROC analysis, suggests the potential of this group for minimally invasive biomarkers in diagnosing PTC. 

In most patients with PTC exhibiting suppressed Tg levels, a significant decrease in *TSHR* methylation levels and miRNA-146b-3p, miRNA-222-3p, and miRNA-221-5p expression was observed postoperatively. The half-life of serum Tg ranges from 1 to 3 days, with the postoperative nadir typically reached in nearly all patients within 3 to 4 weeks [41]. Following I-131 therapy, it can take several months for Tg to be entirely cleared from circulation [42]. Tg measurements can be conducted during thyroid hormone therapy or after TSH stimulation, which is the gold standard for the follow-up of PTC patients [43]. To confirm their prognostic value, it is advisable to observe these changes after one year and continue follow-up assessments for potential recurrence.

Some studies have specifically looked at the correlation between miRNA expression and *TSHR* methylation. For instance, high levels of miR-146a have been correlated with hypermethylation of the *TSHR* promoter and reduced *TSHR* expression in thyroid cancer tissues. Studies have shown that certain miRNAs are differentially expressed in thyroid cancers and autoimmune thyroid diseases, which might correlate with changes in *TSHR* expression. For example, miRNA-155 and miRNA-146a have been implicated in thyroid carcinogenesis and may influence *TSHR* expression [44,45,46]. 

We found a weak positive correlation between the methylation level of *TSHR* and the expression levels of miRNA-146b-3p and miRNA-222-3p before and after surgery. 

Our findings indicate that the plasma *TSHR* methylation and miRNA-146b-3p, -222-3p, and -221-5p expression levels underwent substantial alterations post-thyroidectomy, suggesting their potential role as indicators of complete tumor resection and as valuable prognostic markers. In PTC patients, the high levels of these miRNAs are reflective of their role in maintaining the malignant phenotype. Tumor cells produce and release these miRNAs, leading to elevated levels in the tissue and the bloodstream. After the surgical removal of the tumor, the primary source of these oncomiRs is eliminated. Consequently, the levels of these miRNAs decrease, reflecting a reduction in tumor burden and a response to the surgical intervention. In PTC patients, increased methylation of the *TSHR* gene is a marker of malignant transformation. The methylation status reflects the epigenetic changes that contribute to the tumor’s growth and progression. Following surgery, the removal of the tumor tissue results in a decrease in the overall methylation burden in the remaining thyroid tissue. This decrease indicates the successful resection of the malignant cells that harbored these epigenetic changes.

Our findings indicate a significant association between the expression levels of specific microRNAs and the clinicopathological features of PTC. This study comprehensively analyzed the associations of microRNA expression with the clinicopathological features of papillary thyroid carcinoma (PTC). Significant correlations were observed between the expression levels of specific miRNAs—miRNA-146b-3p, miRNA-222-3p, miRNA-221-5p, and miRNA-21a-3p—and various unfavorable clinical features of PTC, such as extrathyroidal invasion, lymph node metastasis, and tumor multifocality [39]. Numerous studies have previously investigated the roles of miRNA-146b, miRNA-222, miRNA-221, and miRNA-21 in thyroid neoplasia, establishing these miRNAs as both diagnostic and prognostic markers [39]. Our findings align with previously published data highlighting the diagnostic and prognostic potential of miRNAs in thyroid neoplasia. For instance, miRNA-221 and miRNA-222 have been extensively studied in the context of thyroid cancer. Yang Z. et al. demonstrated that miR-221 and miR-222 were overexpressed in PTC and were associated with aggressive clinicopathological features, including extrathyroidal extension and lymph node metastasis [47]. Similarly, Lee J.C. et al. found that elevated levels of miR-222 correlated with tumor aggressiveness and recurrence in PTC patients [48]. MiRNA-21 was upregulated in PTC and was significantly associated with lymph node metastasis and cell proliferation [26,49]. Our observation that miRNA-21a-3p expression is higher in cases of extrathyroidal extension supports these findings and suggests a potential role for miR-21 as a marker of tumor invasiveness. Moreover, patients with PTC who exhibited lymphovascular invasion demonstrated significantly lower expression levels of miRNA-146b-3p and miRNA-21a-3p, with *p*-values of 0.005 and 0.007, respectively. In contrast, our study revealed that plasma levels of miRNA-21 were significantly elevated in cases of PTC with extrathyroidal extension, with a *p*-value of 0.008. However, in the literature, miRNA-146b-3p and miRNA-21a-3p are usually highly expressed in PTCs with more aggressive clinicopathological features [39,50]. These results may offer insights into the molecular mechanisms underlying tumor invasion and spread. On the other hand, lifestyle and environmental exposures, such as smoking, diet, and exposure to toxins, can influence miRNA expression [51]. These factors vary widely among individuals and can significantly impact miRNA profiles in cancer. Other health conditions present in individuals, such as diabetes or heart disease, can influence miRNA expression [52,53]. Analyzing interindividual miRNA variability could help answer the rising questions [54]. Understanding and accounting for these factors is crucial in cancer research to accurately interpret miRNA expression data and their implications in cancer biology and treatment. The extent of intraindividual variability in miRNA levels is a critical question that must be answered. Considering the potential of these miRNAs as independent predictive factors of PTC’s unfavorable features, our data suggest that miR-222-3p and miR-21a-3p could be particularly useful. The elevated levels of miR-222-3p in multifocal PTC and miR-21a-3p in extrathyroidal extension highlight their potential as markers for the aggressiveness of PTC. Overall, our findings contribute to the growing body of evidence supporting the utility of miRNAs as biomarkers of PTC.

Elevated levels of miRNA-222-3p in multifocal PTC may be attributed to its role in regulating genes implicated in cancer progression and metastasis. miRNA-222 functions as an income, promoting oncogenic processes by targeting tumor suppressor genes and the genes involved in cell cycle regulation, apoptosis, and differentiation [55]. Specifically, miRNA-222 downregulates tumor suppressor genes such as *PTEN* [55], p27 [56] and p57 [56,57], and *SLC4A4* [58], resulting in uncontrolled cell proliferation and survival and contributing to tumor growth and multifocality. miRNA-222 is also implicated in regulating epithelial–mesenchymal transition (EMT), a process through which epithelial cells acquire mesenchymal properties, increasing their migratory and invasive capabilities [59]. EMT is a critical step in cancer metastasis [60] and the development of multifocal tumors [61]. Furthermore, miRNA-222 may interact with other signaling pathways such as PI3K/AKT [62], which are involved in the pathogenesis of thyroid cancer. This crosstalk can amplify oncogenic signals and promote multifocality. 

We observed elevated levels of *TSHR* methylation in females compared to males. This finding might have potential implications for treatment decisions and monitoring strategies. Females exhibit higher estrogen levels, impacting gene expression and DNA methylation patterns [62]. Estrogen can modulate the activity of various enzymes involved in the methylation process, potentially leading to increased *TSHR* methylation in females. Furthermore, females possess two X chromosomes, with one undergoing random inactivation, which can influence the expression and methylation of X-linked genes, contributing to sex-specific differences [63]. Variations in diet, lifestyle, and exposure to environmental toxins between males and females can also influence DNA methylation [64]. Hormonal fluctuations associated with pregnancy, childbirth, and breastfeeding can further alter gene expression and methylation patterns [65,66,67]. Epigenetic regulation, including DNA methylation, is governed by a complex interplay of genetic, hormonal, and environmental factors, which can differ between males and females due to their distinct biological and physiological contexts [68]. Further research is needed to elucidate the precise molecular mechanisms underlying *TSHR* methylation, the role of sex hormones, and the impact of genetic and environmental factors on the development and progression of thyroid cancer.

A limitation of our study is its focus on comparing the PTC and HC groups exclusively. A significant challenge in clinical practice is distinguishing between benign nodular goiter and thyroid cancer, which this study does not address. Conducting functional studies could provide a deeper understanding of the biological mechanisms involved. This study does not consider potential confounding factors, such as other underlying health conditions, medications, or lifestyle influences, which could affect miRNA expression and *TSHR* methylation levels. Although the biomarkers show promise, this study does not evaluate their clinical utility and cost-effectiveness for routine clinical practice. 

One more limitation is the lack of immunohistochemistry and miRNA expression analysis in tissue samples. The differences in analyzed miRNA expression and *TSHR* expression between cancerous tissue and normal tissue could help in understanding the biological behavior of the tumor. Moreover, the association between analyzed miRNA expression and *TSHR* expression is important to reveal the possible role of these miRNAs in regulating *TSHR* expression.

While our study provides valuable insights, its small sample size and the absence of tissue sample analysis are notable limitations that may impact the comprehensiveness of the findings.

In conclusion, this study demonstrates the potential utility of miRNAs and *TSHR* methylation levels as biomarkers for diagnosing and monitoring PTC. The preoperative elevation and postoperative reduction in these biomarkers in PTC patients and their higher levels in plasma samples before surgery compared to healthy controls indicate them as candidate biomarkers in the diagnosis and prognosis of PTC. Elevated *TSHR* methylation levels were shown to have a statistically significant weak positive correlation with larger tumor sizes and lymphovascular invasion. Increased miRNA-222-3p levels were associated with tumor multifocality, further emphasizing their clinical relevance. The high AUCs of the investigated miRNAs do not reach 0.8, suggesting only their potential as diagnostic tools for early PTC detection. Nevertheless, further research is essential to validate these biomarkers for broad clinical application, ensuring their effectiveness and reliability across diverse patient populations.

## 4. Materials and Methods

### 4.1. Study Group

Our research involved participants diagnosed with PTC (papillary thyroid carcinoma) and a control group of healthy individuals (HC). Plasma samples were collected from PTC patients treated at the Hospital of Lithuanian University of Health Sciences Kaunas Clinics between 2020 and 2022, both before surgery and four weeks post-surgery. Histological examinations confirmed PTC diagnoses from surgical thyroid tissue samples. PTC patients were staged according to the 8th edition of the AJCC/UICC system [51], and further categorized into aggressive (e.g., diffuse sclerosing variant and tall cell carcinoma) and non-aggressive subtypes (e.g., classical and follicular variants). Excluded from the PTC group were patients with benign thyroid nodules and those with a history of other cancers.

The control group included individuals with no thyroid diseases, autoimmune disorders, or cancer history. All potential participants underwent thyroid ultrasound, hormone tests (TSH and fT4), and anti-thyroid peroxidase (anti-TPO) antibody evaluations before inclusion in the study.

Twelve weeks post-surgery, plasma thyroglobulin (Tg) levels were measured in all PTC patients before they received radioiodine therapy. Tg levels below 0.1 ng/mL were classified as suppressed. This study was approved by the Kaunas Regional Biomedical Research Committee (Lithuania, approval no. BE-2-44; 2015-12-23 and no. BE-2-64; 7 February 2020). All participants provided written informed consent after a detailed explanation of the study’s goals and procedures. This research followed the principles of the Declaration of Helsinki.

### 4.2. DNA Samples

Venous blood samples were collected from both the PTC and HC groups. Peripheral blood (10 mL) was drawn into EDTA tubes (BD Vacutainer PPT™ Plasma Preparation Tube; 13 × 100 mm/5 mL) and centrifuged at 1900× *g* for 10 min at 4 °C. The supernatant was transferred into new conical tubes and centrifuged at 16,000× *g* for 10 min at 4 °C. Purified plasma was aliquoted into 1.5 mL portions and stored at −80 °C until nucleic acid extraction.

### 4.3. DNA and RNA Extraction

Circulating cell-free DNA (cfDNA) was extracted from 5 mL of plasma using the QIAamp Circulating Nucleic Acid Kit (Qiagen, Hilden, Germany) as per the manufacturer’s instructions. The cfDNA was then stored in 0.2 mL Eppendorf tubes at −80 °C.

MicroRNA (miRNA) was extracted from 200 μL of thawed plasma using the miRNeasy Serum/Plasma Kit (Qiagen, Hilden, Germany) with a synthetic spike-in control, Caenorhabditis elegans miRNA-39 (cel-miR-39-3p), added for normalization. Hemolysis was assessed by centrifuging 100 μL of plasma at 1600× *g* for 4 min at 4 °C and measuring oxy-hemoglobin absorbance at 414 nm with a NanoDrop ND1000 Spectrophotometer (ThermoFisher Scientific, Waltham, MA, USA). Samples with OD414 > 0.25 were excluded from analysis.

### 4.4. Bisulfite Conversion and Quantitative Methylation-Specific PCR

For quantitative methylation-specific PCR (QMSP) analysis, up to 400 ng of purified DNA was modified using the EZ DNA Methylation™ Kit (Zymo Research, Irvine, CA, USA) following the manufacturer’s instructions. Samples were incubated at 42 °C for 15 min.

Target-specific QMSP primers and probes were chosen based on previous studies and sourced from Metabion (Martinsried, Germany) (Table 6). ACTB was used as a normalization control. QMSP was performed in duplicate for tissue samples and triplicate for plasma samples. The reaction mixture (20 μL) included TaqMan® Universal Master Mix II, 300 nM of each primer, 50 nM of the probe, and 10 ng of bisulfite-converted DNA. The conditions were as follows: 95 °C for 10 min, 45-50 cycles of 95 °C for 15 s, and 60 °C for 1 min using the QuantStudio 5 Real-Time PCR System (Applied Biosystems™, Waltham, MA, USA). The runs were considered valid if the methylated controls showed positive signals and no amplification in the no template control (NTC). Methylation levels were calculated using the ΔΔCq algorithm and expressed as a percentage of the methylation-positive control.

### 4.5. Quantitative Reverse Transcription–Polymerase Chain Reaction

MiRNA expression levels were quantified using qRT-PCR with the TaqMan Small RNA Assay (Applied Biosystems, Foster City, CA, USA). cDNA was synthesized from extracted RNA using the TaqMan MicroRNA Reverse Transcription Kit. The reaction mix contained 5 ng of RNA, dNTPs, MultiScribe reverse transcriptase, PCR buffer, RT primer, RNase inhibitor, and nuclease-free water, and was cycled at 16 °C for 30 min, 42 °C for 30 min, and 85 °C for 5 min. Specific Thermo Fisher Scientific (Waltham, Massachusetts, USA) primers and probes for miR-21a-3p (accession number MI0000569-000397), miR-221-5p (accession number MI0000298-000524), miR-222-3p (accession number MI0000299-002276), and miR-146b-3p (accession number MI0003129-001097) were used for qPCR on the Rotor-Gene 6000 thermal cycler. The reaction conditions were 95 °C for 10 min, 40 cycles of 95 °C for 15 s, and 60 °C for 60 s. The reaction mix contained TaqMan Universal PCR Master Mix, Small RNA Assay, cDNA, and nuclease-free water. *C. elegans* miRNA-39 was used as an internal control. The fold change in miRNA expression was calculated using the 2^−ΔΔCt^ method. 

### 4.6. Statistical Analysis

The analysis of categorical variables was conducted using the chi-square (χ^2^) test. The normality of continuous variables was assessed using the Kolmogorov–Smirnov test. For non-normally distributed variables, the Mann–Whitney U test was used to compare the two groups. Pearson’s correlation examined the relationships between plasma DNA methylation and quantitative parameters. The diagnostic performance of the biomarkers was assessed using the area under the ROC curve (AUC). Analyses were performed using SPSS 22.0 software, with statistical significance set at *p* < 0.05.

## Figures and Tables

**Table 1:** Demographic and clinicopathological characteristics of the study population with PTC and the control group (HC).

Characteristic	PTC *n* = 46	HC *n* = 57	*p*-Value
Gender			
Male	8 (17.4%)	12 (21.1%)	
Female	38 (82.6%)	45 (78.9%)	
Age at initial surgery (years)	48.6 (14.6)	45.21 (12.9)	*p* = 0.236
T (TNM), *n* (%)		-	-
pT1a	15 (32.6)		
pT1b	4 (8.7)		
pT2	3 (6.5)		
pT3a	15 (32.6)		
pT3b	9 (19.6)		
Tumor size (cm)		-	-
≤2	32 (69.6)		
>2	14 (30.4)		
Lymph node metastases at initial surgery		-	-
Yes	12 (26.1)		
No	34 (73.9)		
Variant of PTC, *n* (%)		-	-
Classical variant	18 (39.1)		
Follicular variant	10 (21.7)		
Diffuse sclerosing variant	15 (32.6)		
Tall cell carcinoma	3 (6.5)		
Extrathyroidal extension		-	-
Yes	24 (52.2)		
No	22 (47.8)		
Lymphovascular invasion		-	-
Yes	25 (54.3)		
No	21 (45.7)		
Multifocality		-	-
Yes	13 (28.3)		
No	33 (71.7)		

**Table 2:** Comparison of miRNA expression levels in plasma samples of PTC patients before surgery and HCs.

miRNA	Relative Expression 2^−ΔΔCt^: Median (IQR)		*p*
	Healthy Controls (*n* = 57)	Preoperative PTC (*n* = 46)	
146b-3p	0.011 (0.009)	0.016 ( 0.027)	0.009
222-3p	0.018 (0.026)	0.038 (0.04)	<0.001
21a-3p	0.072 (0.063)	0.13 (0.21)	<0.001
221-5p	0.007 (0.008)	0.015 (0.013)	0.003

**Table 3:** Plasma miRNA and *TSHR* levels in PTC patients before and after thyroidectomy.

miRNA	Relative Expression 2^−ΔΔCt^: Median (IQR) *TSHR* Methylation Levels: Median (IQR)		*p*
	Preoperative PTC (*n* = 46)	Postoperative PTC (*n* = 46)	
146b-3p	0.016 (0.027)	0.012 (0.011)	0.009
222-3p	0.038 (0.04)	0.022 (0.016)	<0.001
21a-3p	0.13 (0.21)	0.069 (0.08)	0.003
221-5p	0.015 (0.013)	0.009 (0.007)	<0.001
***TSHR* methylation**	26.041 (18.740)	21.382 (16.220)	<0.001

**Table 4:** Correlation between clinicopathological features of PTC; miRNA-146b, miRNA-222-3p, miRNA-21a-3p, and miRNA-221-5p expression levels; and *TSHR* methylation levels in plasma samples. * *p* < 0.05.

PTC Clinicopathological Feature	Relative Expression 2^−ΔΔCt^: Median (IQR)				DNA Methylation Level: Median (IQR)
	miRNA-146b-3p	miRNA-222-3p	miRNA-21a-3p	miRNA-221-5p	*TSHR*
**Age**					
<55 years (*n* = 30; 68.2%)	0.017 (0.026)	0.038 (0.045)	0.13 (0.17)	0.014 (0.013)	30.41(18.78)
≥55 years (*n* = 16; 33.8%)	0.018 (0.034)	0.038 (0.021)	0.10 (0.24)	0.016 (0.015)	24.09 (10.99)
*p*	0.908	0.963	0.729	0.344	0.219
**Gender**					
Male (*n* = 8; 16.3%)	0.021 (0.036)	0.035 (0.033)	0.079 (0.256)	0.017 (0.015)	24.09 (12.01)
Female (*n* = 38; 83.7%)	0.017 (0.246)	0.04 (0.041)	0.134 (0.184)	0.013 (0.014)	30.88 (21.32)
*p*	0.701	0.831	0.467	0.201	0.038 *
**Multifocality**					
Single (*n* = 13; 28.3%)	0.015 (0.018)	0.032 (0.035)	0.103 (0.152)	0.013 (0.013)	30.39 (20.13)
Multiple (≥2) (*n* = 33; 71.7%)	0.033 (0.051)	0.061 (0.046)	0.232 (0.383)	0.018 (0.013)	25.04 (13.01)
*p*	0.154	0.016 *	0.147	0.329	0.259
**Extrathyroidal extension**					
Yes (*n* = 24; 71.4%)	0.025 (0.027)	0.028 (0.04)	0.079 (0.112)	0.013 (0.009)	26.04 (11.80)
No (*n* = 22; 28.6%)	0.013 (0.02)	0.045 (0.04)	0.199 (0.169)	0.0162 (0.013)	27.07 (23.00)
*p*	0.09	0.132	0.008 *	0.132	0.856
**Lymphovascular invasion**					
Yes (*n* = 24; 52.2%)	0.011± 0.017	0.031 (0.04)	0.085 (0.119)	0.012 (0.013)	30.41 (19.40)
No (*n* = 22; 48.8%)	0.03 (0.028)	0.049 (0.042)	0.207 (0.131)	0.016 (0.013)	23.99 (14.78)
*p*	0.005 *	0.087	0.007 *	0.147	0.039 *
**T (TNM)**					
T1a, T1b (*n* = 19; 20.6%)	0.018 ( 0.027)	0.046 (0.042)	0.192 (0.21)	0.016 (0.013)	29.34 (23.00)
T2, T3 (*n* = 27; 79.4%)	0.016 (0.26)	0.034 (0.035)	0.088 (0.1840	0.014 (0.011)	26.04 (11.80)
*p*	0.289	0.220	0.051	0.326	0.816
**Tumor size (cm)**					
≤2 (*n* = 32; 69.6%)	0.017 (0.029)	0.038 (0.046)	0.134 (0.164)	0.014 (0.013)	24.23 (14.18)
>2 (*n* = 14; 30.6%)	0.018 (0.019)	0.036 (0.030)	0.087 (0.12)	0.015 (0.013)	32.64 (27.58)
*p*	0.402	0.272	0.733	0.412	0.002 *
**Lymph node metastases (preoperative PTC plasma samples)**					
Yes (*n* = 12; 25%)	0.026 (0.056)	0.034 ( 0.039)	0.136 (0.372)	0.013 (0.016)	26.04 (17.86)
No (*n* = 34; 75%)	0.015 (0.027)	0.04 ( 0.056)	0.14 (0.208)	0.013 (0.014)	27.07 (19.92)
*p*	0.317	0.591	0.764	0.548	0.490

**Table 5:** An analysis of ROC curves to determine the efficacy of plasma expression levels of miRNA-146b-3p, miRNA-21a-3p, miRNA-221-5p, and miRNA-222-3p and the methylation levels of *TSHR* in discriminating between patients with PTC and HC. * *p* < 0.05.

miRNA	AUC	Asymptotic 95% CI		*p*	miRNA’s Expression 2^−ΔΔCt^, *TSHR* Methylation Level Cut-Offs	Sensitivity	Specificity
		Lower Bound	Upper Bound				
146b-3p	0.651	0.541	0.761	0.007 *	0.0126	65.2%	33.33%
222-3p	0.708	0.609	0.807	<0.0001 *	0.0143	87.9%	52.6%
21a-3p	0.673	0.566	0.779	0.001 *	0.1300	52.2%	17.5%
221-5p	0.765	0.674	0.855	<0.0001 *	0.0103	66.0%	71.7%
***TSHR* methylation levels**	0.719	0.620	0.818	<0.0001 *	16.4960	86.7%	63.2%

**Table 6:** *TSHR* and *ACTB* primers and probes.

Gene Symbol	DNA Strand	Primer ID	Primer Sequence (5′→3′)	Amplicon Size, bp
***TSHR***	Sense	Fwd	GGTGTAGAGTTGAGAATGAGGTGATTTC	122
		Rev	GCCCAAATCCCTAAACAAATCG	
		Probe	FAM-ACAACACCAACTACAACAAATCCGCCGA-BHQ1	
***ACTB***	Antisense	Fwd	TGGTGATGGAGGAGGTTTAGTAAGT	133
		Rev	AACCAATAAAACCTACTCCTCCCTTAA	
		Probe	FAM-ACCACCACCCAACACACAATAACAAACACA-BHQ- 1	

## Data Availability

Data are contained within the article.
